# Kinetics of Neutralizing Antibodies against Omicron Variant in Vietnamese Healthcare Workers after Primary Immunization with ChAdOx1-S and Booster Immunization with BNT162b2

**DOI:** 10.4269/ajtmh.22-0434

**Published:** 2022-11-30

**Authors:** Nguyen Van Vinh Chau, Lam Anh Nguyet, Nguyen Thanh Dung, Vo Minh Quang, Nguyen Thanh Truong, Le Mau Toan, Le Manh Hung, Dinh Nguyen Huy Man, Dao Bach Khoa, Nguyen Thanh Phong, Nghiem My Ngoc, Huynh Phuong Thao, Dinh Thi Bich Ty, Pham Ba Thanh, Nguyen Thi Han Ny, Le Kim Thanh, Cao Thu Thuy, Nguyen To Anh, Nguyen Thi Thu Hong, Le Nguyen Truc Nhu, Lam Minh Yen, Guy Thwaites, Tran Tan Thanh, Le Van Tan, for OUCRU COVID-19 Research Group

**Affiliations:** ^1^ Department of Health, Ho Chi Minh City, Vietnam;; ^2^ Centre for Tropical Medicine and Global Health, Nuffield Department of Medicine, University of Oxford, Oxford, United Kingdom;; ^3^ Oxford University Clinical Research Unit, Ho Chi Minh City, Vietnam;; ^4^ Hospital for Tropical Diseases, Ho Chi Minh City, Vietnam;; ^5^ Tan Phu Hospital, Ho Chi Minh City, Vietnam

## Abstract

We studied the development and persistence of neutralizing antibodies against SARS-CoV-2 ancestral strain, and Delta and Omicron (BA.1 and BA.2) variants in Vietnamese healthcare workers (HCWs) up to 15 weeks after booster vaccination. We included 47 HCWs, including group 1 (G1, *N* = 21) and group 2 (G2; *N* = 26) without and with breakthrough Delta variant infection before booster immunization, respectively). The study participants had completed primary immunization with ChAdOx1-S and booster vaccination with BNT162b2. Neutralizing antibodies were measured using a surrogate virus neutralization assay. Of the 21 study participants in G1, neutralizing antibodies against ancestral strain, Delta variant, BA.1, and BA.2 were (almost) abolished at month 8 after the second dose, but all had detectable neutralizing antibodies to the study viruses at week 2 post booster dose. Of the 26 study participants in G2, neutralizing antibody levels to BA.1 and BA.2 were significantly higher than those to the corresponding viruses measured at week 2 post breakthrough infection and before the booster dose. At week 15 post booster vaccination, neutralizing antibodies to BA.1 and BA.2 dropped significantly, with more profound changes observed in those without breakthrough Delta variant infection. Booster vaccination enhanced neutralizing activities against ancestral strain and Delta variant compared with those induced by primary vaccination. These responses were maintained at high levels for at least 15 weeks. Our findings emphasize the importance of the first booster dose in producing cross-neutralizing antibodies against Omicron variant. A second booster to maintain long-term vaccine effectiveness against the currently circulating variants merits further research.

## INTRODUCTION

COVID-19 vaccine induced immunity wanes,^1^^,^^2^ which has led to the administration of booster doses worldwide. Follow-up studies assessing the impact of booster vaccination on the development and persistence of the immune response to SARS-CoV-2 and circulating variants of concern (VOC) remain critical to informing the allocation of resources, policy decisions on COVID-19 mitigation measures, and the development of next-generation vaccines.^3^

Over the past 12 months, SARS-CoV-2 Delta and Omicron VOCs have been responsible for two consecutive COVID-19 waves globally. Omicron variant is genetically divided into five major sublineages: BA.1–5. Earlier in 2022, BA.2 replaced BA.1 to become the dominant variant worldwide, including in Vietnam.^4^ As of June 13, 2022, BA.4 and BA.5 were responsible for the most recent waves in South Africa and Portugal,^5^^,^^6^ with spread reported into Europe and the United States.^7^

It is thus critical to assess levels of neutralizing antibodies induced by primary and booster vaccination against Delta and Omicron variants, especially in individuals with different preexisting immunity—for example, breakthrough and nonbreakthrough infection. Yet most of the reported data have been from high-income countries,^3^^,^^8^^–^^13^ and few studies, especially those focusing on long term immunity, have been conducted in low- and middle-income countries.

Vietnam started the national COVID-19 vaccination program in March 2021 and introduced the first boosters in December 2021. Diverse vaccine products have been used in Vietnam, including mRNA (BNT162b2 and mRNA-1273), adenoviral vector (Oxford-AstraZeneca (ChAdOx1-S) and SputnikV), whole-inactivated virus (SinoPharm, Hayat-Vax and Covaxin) and protein subunit (Abdala) vaccines. As of August 14, 2022, a total of 251,456,299 doses have been administered, with BNT162b2 and ChAdOx1-S vaccines accounting for 67.2%.^14^ Herein, we focused our analysis on healthcare workers (HCWs) of the Hospital for Tropical Diseases (HTD) in Ho Chi Minh City, Vietnam. Our aim was to assess the impact of the heterologous booster on the development and persistence of neutralizing antibodies against the ancestral strain, Delta, and Omicron variants (BA.1 and BA.2) in HTD staff with and without prior breakthrough infection.

## MATERIALS AND METHODS

### Setting and the vaccine evaluation study.

The present study has been conducted at HTD in Ho Chi Minh City since March 2021.^15^ HTD is a 550-bed tertiary referral hospital for patients with infectious diseases in southern Vietnam. HTD has ∼900 members of staff and has been responsible for receiving COVID-19 patients of all severities in Southern Vietnam since the beginning of the pandemic.

The detailed descriptions about the study cohort have previously been reported.^15^ In brief, a total of 554 individuals were enrolled at baseline, and 144 were selected for followed up from the second dose onward. Two doses of Oxford-AstraZeneca COVID-19 vaccine (ChAdOx1-S) were given as part of the primary course, completed by the first week of May 2021. And Pfizer-BioNTech COVID-19 vaccine (BNT162b2) was given as part the booster dose, completed in the third week of December 2021.

### Weekly SARS-CoV-2 testing.

As per the national COVID-19 control strategy in Vietnam, between June 2021 and March 2022, HTD staff were tested weekly for SARS-CoV-2 using either polymerase chain reaction (PCR) or antigen tests.^16^ When available, samples were subjected to SARS-CoV-2 whole-genome sequencing to determine SARS-CoV-2 variant.^17^ This allowed for the detection of breakthrough infection. We previously reported a cluster of breakthrough Delta variant infection among HTD staff members in June 2021.^18^ Any staff members with breakthrough infection requiring hospitalization was admitted to HTD for clinical care.

### Plasma samples for antibody measurement.

**Figure 1.**
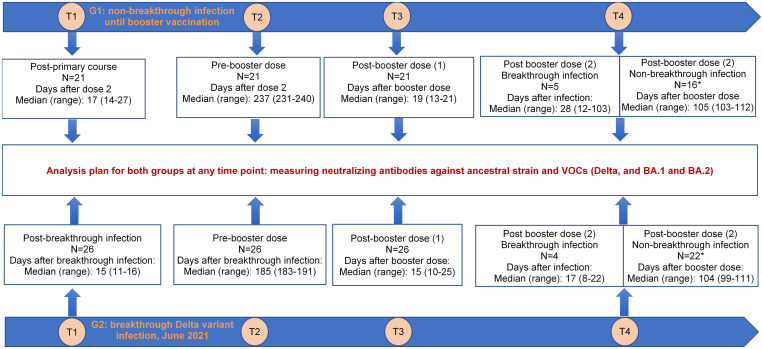
Illustration showing the distribution of the study participants and sampling schedules for neutralizing antibody measurement. * After excluding cases with a SARS-CoV-2 infection episode recorded after the booster dose.

We selected 47 HCWs from the original vaccine evaluation study, consisting of group 1 (G1) including 21 without documented breakthrough infection from baseline until booster vaccination, and group 2 (G2), including 26 with breakthrough Delta variant infection.^15^ More detailed descriptions about the selected participants and sampling schedules are presented in Figure 1.

### Sample size justification.

A total of 47 HCWs were selected for analysis. Although the selected sample size was pragmatic in nature and was dependent on the availability of the resources, sampling at this scale has been proven to be sufficient to demonstrate the differences in antibody responses to Omicron variant in people receiving either heterologous or homologous BNT162b2 booster vaccination.^12^ Additionally, these individuals were selected because they had longitudinal plasma samples collected from dose 2 (G1) or breakthrough infection (G2) until month 3 after the booster dose, thus also allowing for assessment of the kinetics of neutralizing antibody titers at individual level.

### Antibody measurements.

For measurement of neutralizing antibodies against SARS-CoV-2 original strain (herein referred as ancestral) and SARS-CoV-2 Delta and Omicron variants (BA.1 and BA.2), we used the SARS-CoV-2 Surrogate Virus Neutralization (sVNT) assay (catalog no. L00847; Genescript, Piscataway, NJ). sVNT is a blocking ELISA that quantifies neutralizing antibodies targeting the receptor binding domain of S protein.^19^ The experiments were carried out as per the manufacturer’s instructions with the readouts expressed as percentage of inhibition.

### Statistical analysis.

The Wilcoxon signed-rank test or the paired *t*-test or Mann–Whitney *U* test was used to compare the differences in neutralizing antibody levels to ancestral strain, Delta, BA.1, and BA.2 between and within groups when appropriate. The Spearman’s correlation was used to assess the correlation of neutralizing antibody levels and age. All analyses were performed using GraphPad Prism 9.3.1 (GraphPad Software, La Jolla, CA).

### Ethics.

The study received approvals from the Institutional Review Board of the HTD in Ho Chi Minh City Vietnam and the Oxford Tropical Research Ethics Committee. Written informed consent was obtained from all the study participants.

## RESULTS

### Demographics and breakthrough infection after booster vaccination.

Information about the demographics and vaccination status of the selected participants are presented in Table 1 and Figure 1. The window time between the second dose and the booster dose was ∼8 months. Of the 26 participants in G2, the window time from infection to booster vaccination was ∼6 months (Figure 1).

**Table 1:** Demographics and time intervals between vaccine doses

Variables	G1: HCWs without documented breakthrough infection prior to booster vaccination (*N* = 21)	G2: HCWs with breakthrough infection prior to booster vaccination (*N* = 26)
Male gender, n (%)	1 (4.8)	10 (38.5)
Age year, median (range)	35 (24–54)	40.5 (24–56)
Vaccine dose 1 date (range)	March 8–12, 2021	March 8–15, 2021
Vaccine dose 2 date (range)	April 19–28, 2021	April 22–May 4, 2021
Vaccine dose 3 date (range)	December 16–17, 2021	December 16–21, 2021
Days from vaccine dose 1 to dose 2, median (range)	43 (40–49)	44 (39–53)
Days from vaccine dose 2 to dose 3, median (range)	238 (233–241)	238 (231–245)
Days from vaccine dose 3 to breakthrough infection, median (range)	NA	187 (184–191)
Breakthrough infection after booster dose, n (%)*	5 (23.8)	4 (15.4)
Days from booster vaccination to infection, median (range)	82 (1–94)	86 (77–96)
Days from infection after booster dose to blood sampling, median (range)	28 (12–103)	17 (8–22)

HCW = healthcare worker; NA = nonapplicable.

* The figure refers to the time interval between the booster dose and the day that the participant tested positive by polymerase chain reaction or rapid test.

During the follow-up, 9 individuals, including 5 or 21 (24%) participants of G1 and 4 of 26 (15%) participants of G2, had a SARS-CoV-2 infection episode recorded after the booster dose (Figure 1). Although detailed clinical descriptions were not available, no hospitalization was reported, suggesting that all were either asymptomatic or mildly symptomatic. E-gene real-time PCR Ct values were available in two samples of G1 (13.1 and 15.4). Of these, information about SARS-CoV-2 variant was available in one, which was assigned to BA.2. The window time (median in days) from infection to blood sampling at month 3 after booster vaccination was 28 (range: 12–103) for G1 and 17 (range: 8–22) for G2.

### Neutralizing antibodies against BA.1 and BA.2 after primary vaccination with ChAdOx1-S.

**Figure 2.**
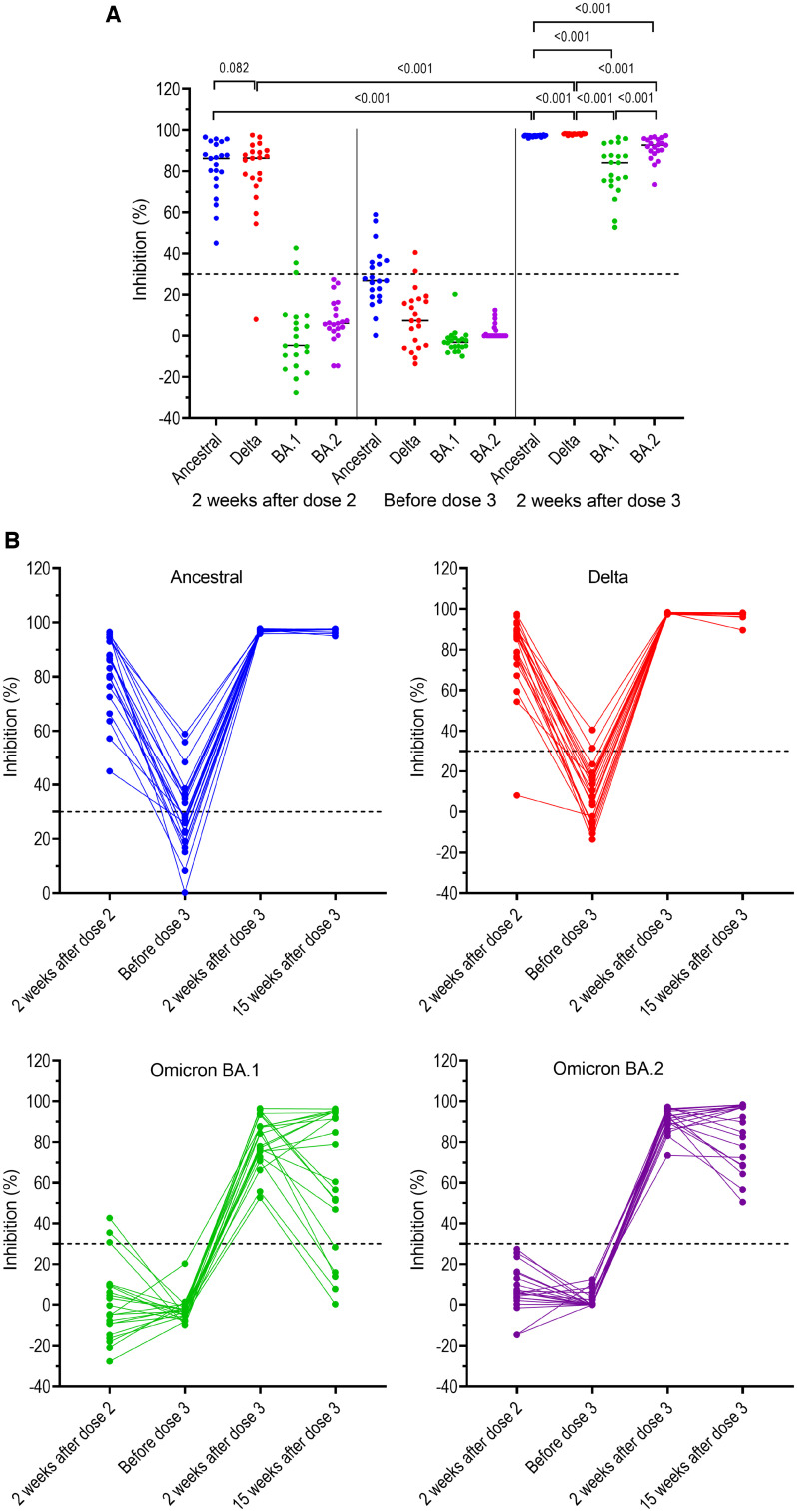
Neutralizing antibodies against SARS-CoV-2 ancestral strain and variants of concerns (VOCs) (Delta, BA.1, and BA.2) in individuals without prior breakthrough Delta variant infection measured at different time points prior to booster vaccination and at week 2 post booster dose. (**A**) Back-to-back comparison between neutralizing antibody levels against VOCs. (**B**) Kinetics of neutralizing antibodies for individual participants. Horizontal dot lines indicate assay cut-off. Numbers indicate *P* values.

Of the 21 participants in G1, at week 2 after the primary course, detectable neutralizing antibodies against ancestral strain and Delta variant were documented in 21 (100%) and 20 (95%), with comparable levels to ancestral strain and Delta variant (Figure 2A and B, Table 2). Neutralizing antibodies to BA.2 were not detected, whereas neutralizing antibodies against BA.1 were detected in only three participants but the titers approached the detection limit of the sVNT assay (Figure 2A and B, Table 2).

**Table 2:** Neutralizing antibody levels to ancestral strain, Delta variant, BA.1, and BA.2 measured at four time points during the study period

		Ancestral strain	Delta variant	BA.1	BA.2
G1: HCWs without prior BI	2 weeks after D2	86.2 (74.6–93.8)	86.3 (74.4–89.8)	−4.7 (–12 to 9.5)	6.1 (2.9–14.4)
	Before D3	26.9 (19.1–36.2)	7.5 (–5.3–17.5)	−3.2 (–5.5 to –1)	0 (0–3.3)
	2 weeks after D3	97.3 (96.9–97.5)	98.2 (98.0–98.3)	84.1 (74.1–90.6)	92.7 (89.2–95.6)
	15 weeks after D3	97.5 (97.4–97.6)	97.9 (97.4–98.1)	54.3 (19.1–92.1)	83.7 (68.3–97.6)
G2: HCWs with prior BI	2 weeks after BI	96.9 (81.6–97.0)	97.6 (77.2–98.2)	70.0 (10.9–80.8)	85.7 (–5.2 to 92.4)
	Before D3	96.0 (87.8–96.6)	97.5 (71.9–98)	18.8 (6–46.7)	74.1 (34.5–82.9)
	2 weeks after D3	96.1 (95.8–97.5)	98.1 (98.0–98.4)	92.3 (82.8–95.7)	95.7 (90.8–97.7)
	15 weeks after D3	96.8 (96.7–97.0)	98 (98.0–98.2)	87.8 (70.5–93.2)	94.9 (89.0–98.2)

BI = breakthrough infection; D2 = dose 2; D3 = dose 3; HCW = healthcare worker. Reported values are median inhibition in % (interquartile range).

### Development of neutralizing antibodies after heterologous booster with BNT162b2 in individual with and without prior breakthrough infection.

Of the 21 participants in G1, before the booster dose (i.e., month 8 after dose 2), none had detectable neutralizing antibodies against Omicron variant (BA.1 and BA.2). The proportions of individuals with detectable neutralizing antibodies to the ancestral strain and Delta variant were 8/21 (38%) and 2/21 (10%), respectively, with neutralizing titers approaching the assay detection limit (Figure 2A and B). At week 2 after the booster dose, all had neutralizing antibodies against ancestral strain and VOCs (Delta, BA.1, and BA.2). Notably, neutralizing antibody levels to ancestral strain, and Delta variant measured at 2 weeks after the booster dose were significantly higher than those to the respective viruses measured at week 2 after dose 2, described as median inhibition in % (interquartile range [IQR]): for ancestral strain: 97.3 (96.9–97.5) versus 86.2 (74.6–93.8), *P* < 0.001, and for Delta variant: 98.2 (98.0–98.3) versus 86.3 (74.4–89.8), *P* < 0.001) (Figure 2). Neutralizing antibody levels to BA.2 were significantly higher than those to BA.1, median inhibition in % (IQR): 92.7 (89.2–95.6) versus 84.1 (74.1–90.6) (*P* < 0.001; Figure 2A, Table 2).

**Figure 3.**
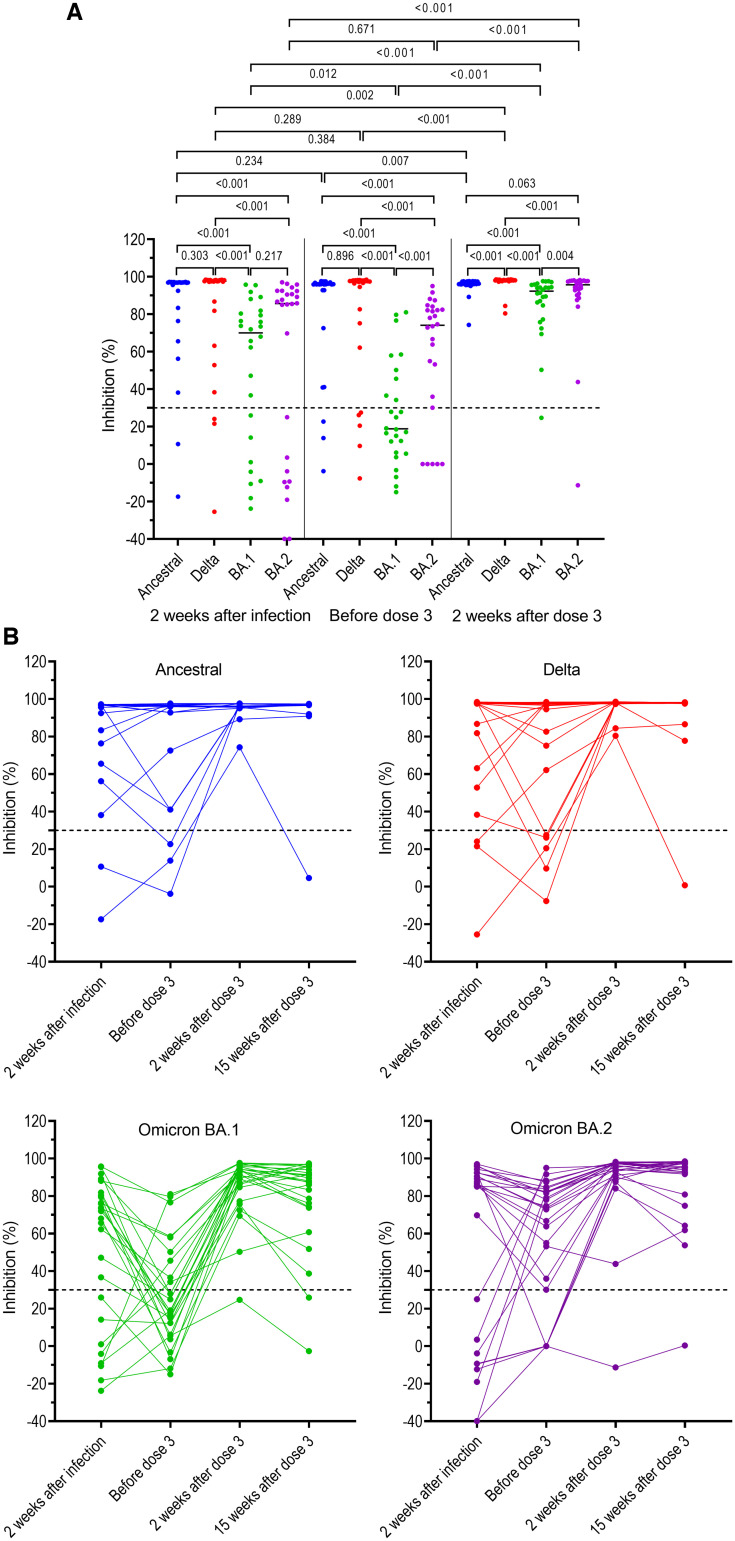
Neutralizing antibodies against SARS-CoV-2 ancestral strain and variants of concerns (VOCs) (Delta, BA.1, and BA.2) in individuals with prior breakthrough Delta variant infection measured at different time points before booster vaccination and at week 2 post booster dose. (**A**) Back-to-back comparison between neutralizing antibody levels against VOCs. (**B**) Kinetics of neutralizing antibodies for individual participants. Horizontal dot lines indicate assay cut-off. Numbers indicates *P* values.

Of the 26 participants in G2, neutralizing antibodies against ancestral strain, Delta, BA.1, and BA.2 measured at week 2 after breakthrough infection were detectable in 24 (92%), 23 (88%), 18 (69%), and 17 (65%), respectively (Figure 3), with neutralizing antibody levels to the ancestral strain and Delta variant significantly higher than those to BA.1 and BA.2 (Figure 3, Table 2). At week 2 after booster vaccination, neutralizing antibody levels to BA.1 and BA.2 significantly increased compared with those measured before the booster dose and at 2 weeks post breakthrough infection but remained significantly lower than those against ancestral strain and Delta variant (Figure 3, Table 2). At this time point, neutralizing antibody levels to BA.2 were significantly higher than those to BA.1, median inhibition in % (IQR): 95.7 (90.8–97.7) versus 92.3 (82.8–95.7) (*P* < 0.004; Figure 3).

### Persistence of neutralizing antibodies at week 15 after booster vaccination.

**Figure 4.**
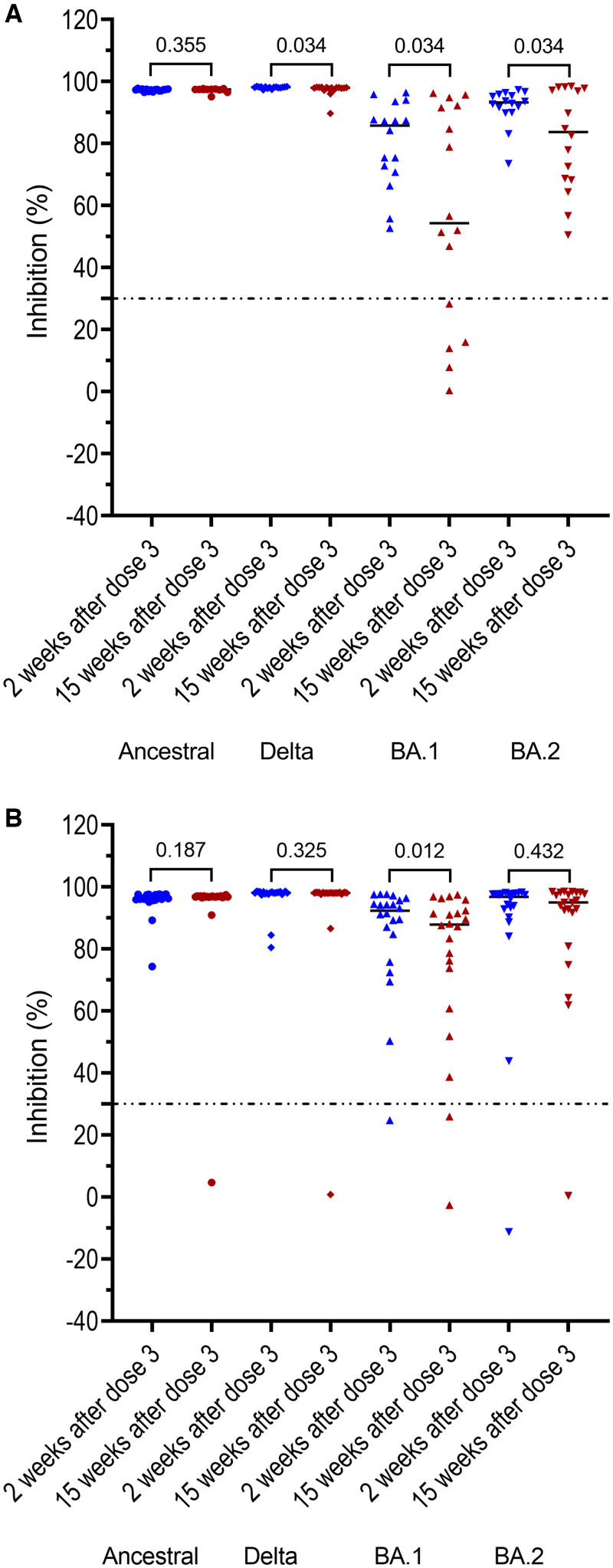
Persistence of neutralizing antibodies at month 3 after the booster dose in those without documented breakthrough infection after the booster dose. (**A**) participants of G1, and (**B**) participants of G2. Horizontal dot lines indicate assay cutoff. Numbers indicates *P* values.

To assess the persistence of neutralizing antibodies induced by the booster dose, we first focused our analysis on those without a SARS-CoV-2 infection episode documented after booster vaccination. At week 15 after the booster dose, of 16 study participants in G1, 16 (100%) had detectable neutralizing antibodies against ancestral, Delta, and BA.2 variants, whereas 11 of 16 (69%) had detectable neutralizing antibodies against BA.1. Accordingly, neutralizing antibody levels to BA.1 and BA.2 was significantly lower compared with those measured at week 2 post booster vaccination, median inhibition in % (IQR): for BA.1: 54.3 (19.1–92.1) versus 85.7 (71.3–92.1), *P* = 0.034, and for BA.2: 83.7 (68.3–97.6) versus 93.2 (90.4–95.7) (*P* = 0.034; Figure 4A). Neutralizing antibodies against Delta variant also slightly reduced but remained at very high titers (Figure 4A).

Of the 22 participants without a SARS-CoV-2 infection episode documented after the booster dose in G2, neutralizing antibody levels to BA.1 significantly decreased, median inhibition in % (IQR): 87.8 (70.5–93.2) versus 92.3 (82.5–95.8) (*P* = 0.012; Figure 4B). Otherwise, neutralizing antibody levels to ancestral strain, Delta and BA.2 measured at this time points were comparable with those of the corresponding viruses measured at two weeks post booster dose (Figure 4B, Table 2).

Of the participants without breakthrough infection documented after the booster dose, back-to-back comparison showed comparable neutralizing antibody levels measured at week 15 after booster immunization between participants of G1 and G2 (Supplemental Figure 1A, Table 2). However, neutralizing antibody levels measured at two weeks after booster vaccination were slightly higher in those with prior Delta variant infection (Supplemental Figure 1B, Table 2).

Of the nine individuals with a documented SARS-COV-2 infection episode after the booster dose, neutralizing antibody levels to ancestral strain and all VOCs slightly increased at week 15, albeit not statistically significant in case of ancestral strain, BA.1 and BA.2 (Supplemental Figure 2).

### Association between age and neutralizing antibody levels to BA.1 and BA.2.

Results of linear regression analysis showed no association between age and neutralizing antibodies levels to BA.1 and BA.2 measured at weeks 2 and 15 post booster dose (Supplemental Figure 3). Similar analysis for ancestral strains and Delta variant was considered uninformative because neutralizing antibody levels to these two viruses in all study participants reached the upper detection limit of the assay (100%) (Figure 3).

## DISCUSSION

We showed that neutralizing antibodies induced by primary immunization with ChAdOx1-S in Vietnamese HCWs failed to neutralize Omicron variant BA.1 and BA.2. Heterologous booster vaccination with BNT162b2 improved the immunity that could broadly neutralize both BA.1 and BA.2 in HCWs with and without prior breakthrough infection. Additionally, booster vaccination significantly enhanced neutralizing antibody levels to the ancestral strain and Delta variant. However, neutralizing antibodies against BA.1 and BA.2 significantly declined at month 3 post-booster vaccination, with comparable levels between participants of G1 and G2. Neutralizing antibodies to ancestral strain and Delta variant remained at high titers over 3 months. We found no association between age and neutralizing antibody levels, in line with a recent report,^11^ but none of our study participants were older than 57 years. Our findings are consistent with existing data regarding the capacity of the Omicron variant to escape from neutralizing antibodies induced by vaccination.^9^^,^^20^ The results also support previous findings about the effectiveness of the third doses in preventing infection, severe disease and death.^13^

More than half of the plasma samples collected at 2 weeks after breakthrough Delta variant infection cross-neutralized BA.1 and BA.2, supporting recent reports regarding protection against Omicron offered by previous infection.^21^^–^^23^ Booster vaccination further enhanced the cross-neutralizing activities and the proportion of plasma samples with detectable neutralizing antibodies in these individuals with breakthrough Delta variant infection 6 months earlier.^18^ Because neutralizing antibodies titers are well correlated with protection,^24^^,^^25^ the data suggest that booster vaccination could still be beneficial to individuals with breakthrough infection in protecting against Omicron variant.^21^^,^^23^

The decline in neutralizing antibody levels to sublineages BA.1 and BA.2 observed at week 15 after the first booster dose is in agreement with a previous report.^11^ Our results also complement findings from a recent population-based study in the United States,^2^ which showed that during the Omicron wave, vaccine effectiveness against hospitalizations dropped from 91% during the first 2 months to 78% ≥ 4 months after a third dose. Additionally, a recent study from Israel demonstrated that a second booster dose of the BNT162b2 vaccine was effective in reducing the risk of COVID-19 associated outcomes (including infection) in individuals already completing the first booster dose at least 4 months earlier.^3^

Our study consistently showed that neutralizing antibody titers against BA.2 after the booster dose in individuals with and without prior breakthrough infection were significantly higher than those against BA.1. Relevant data from previous studies have so far been inconsistent. Recent studies from Germany and Hong Kong showed comparable serum neutralizing antibody levels to BA.1 and BA.2 in individuals completing three doses of BNT162b2.^8^^,^^26^ In contrast, Yu and colleagues showed that median neutralizing antibody titers against BA.2 was lower than those against BA.1 in people triple vaccinated with BNT162b2, and in those with previous infection regardless of the vaccination status.^10^ Within the spike protein, the target of antibodies induced by vaccination or infection, Omicron BA.1 and BA.2 sublineages share 21 amino acid mutations with additional 12 unique mutations in BA.1 and 8 in BA.2.^27^ The differences in spike mutation profiles, study populations, and preexisting immunity induced by past exposure and/or vaccination might be contributing factors. Whether BA.2 is less able to evade immunity than BA.1 merits further research.

Our study has several limitations. First, we did not perform live virus neutralization assay, currently the gold standard, to measure neutralizing antibodies. However, the percentage of inhibition measured by the sVNT test has been shown to correlate well with the neutralizing antibody titers measured by the conventional plaque reduction neutralization assay.^19^ Second, we did not study T-cell responses, which have been proven to play an important role in protecting against severe disease and death, and in case of Omicron variant, despite the neutralization escape, T-cell responses were preserved at ∼70% to 80%.^28^^,^^29^

In summary, we showed that booster vaccination by BNT162b2 induced cross-neutralizing activities against sublineages BA.1 and BA.2 of Omicron variant in Vietnamese HCWs completing primary immunization with ChAdOx1-S. These responses however significantly reduced at month 3 post booster doses. A second booster to maintain long-term vaccine effectiveness against the currently circulating variants merits further research. Vaccines remain critical to reduce the transmission and to protect against severe disease and death.

## Supplemental files


Supplemental materials
