# Supplementary material for: Kinetics of Neutralizing Antibodies against Omicron Variant in Vietnamese Healthcare Workers after Primary Immunization with ChAdOx1-S and Booster Immunization with BNT162b2
Source: Am J Trop Med Hyg. 2022 Nov 30;108(1):137–44. doi: 10.4269/ajtmh.22-0434 (PMC9833090; doi:10.4269/ajtmh.22-0434)
Supplement: Supplementary file 1 [file tpmd220434.SD1.pdf]

Supplementary Materials For

Kinetics of neutralizing antibodies against Omicron variant in Vietnamese healthcare workers after primary immunization with ChAdOx1-S and booster immunization with BNT162b2

Nguyen Van Vinh Chau[1,2] Lam Anh Nguyet[3], Nguyen Thanh Dung[4], Vo Minh Quang[4], Nguyen Thanh Truong[5], Le Mau Toan[4], Le Manh Hung[4], Dinh Nguyen Huy Man[4], Dao Bach Khoa[4], Nguyen Thanh Phong[4], Nghiem My Ngoc[4], Huynh Phuong Thao[4], Dinh Thi Bich Ty[4], Pham Ba Thanh[4], Nguyen Thi Han Ny[3], Le Kim Thanh[3], Cao Thu Thuy[3], Nguyen To Anh[3], Nguyen Thi Thu Hong[3], Le Nguyen Truc Nhu[3], Lam Minh Yen[3], Guy Thwaites[2,3], Tran Tan Thanh[3], and Le Van Tan[2,3], for OUCRU COVID-19 Research Group*

[1]Department of Health, Ho Chi Minh City, Vietnam

[2]Centre for Tropical Medicine and Global Health, Nuffield Department of Medicine, University of Oxford, Oxford, UK

[3]Oxford University Clinical Research Unit, Ho Chi Minh City, Vietnam

[4]Hospital for Tropical Diseases, Ho Chi Minh City, Vietnam

[5]Tan Phu Hospital, Ho Chi Minh City, Vietnam

*Members of the groups are listed in the acknowledgments.

Correspondence: Nguyen Van Vinh Chau, chaunvv@oucru.org, and Le Van Tan, tanlv@oucru.org

Word count: **abstract**: 250 word, **Main text**: 2557 words

Running title: Neutralization of SARS-CoV-2 Omicron variant

Keywords: COVID-19, SARS-CoV-2, Omicron, vaccine, pandemic, Vietnam

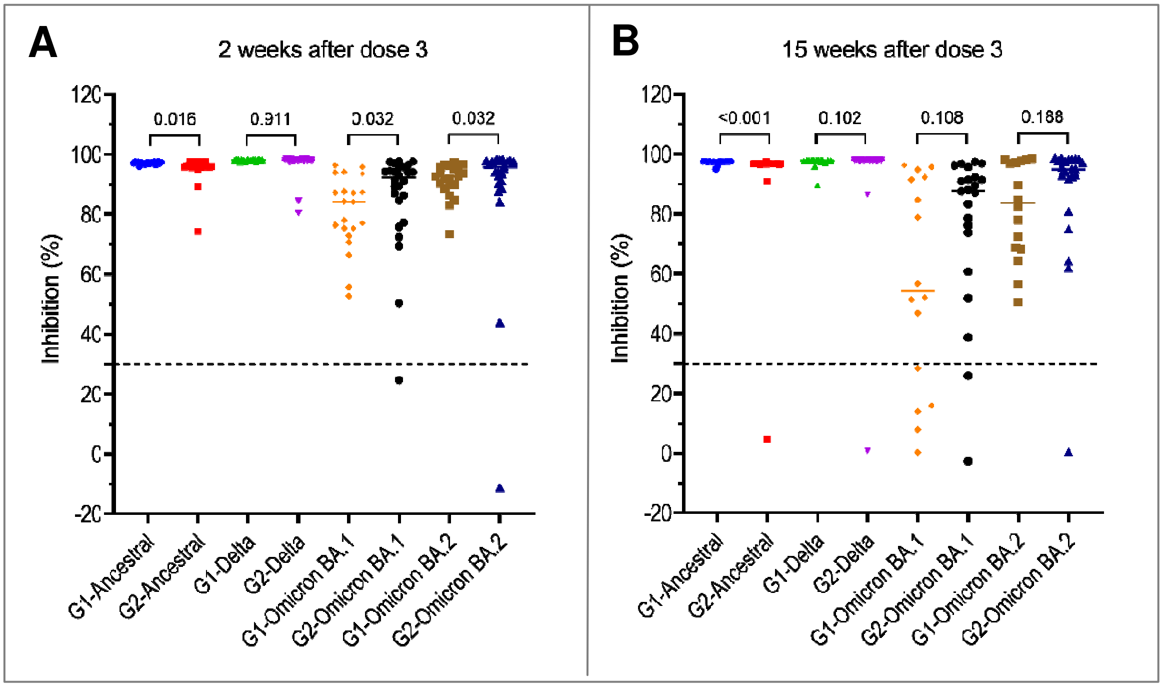

Supplementary Figure 1: Comparison between neutralizing antibody levels obtained from participants of G1 and G2 at 2 (panel A) and 15 (panel B) weeks after booster vaccination.

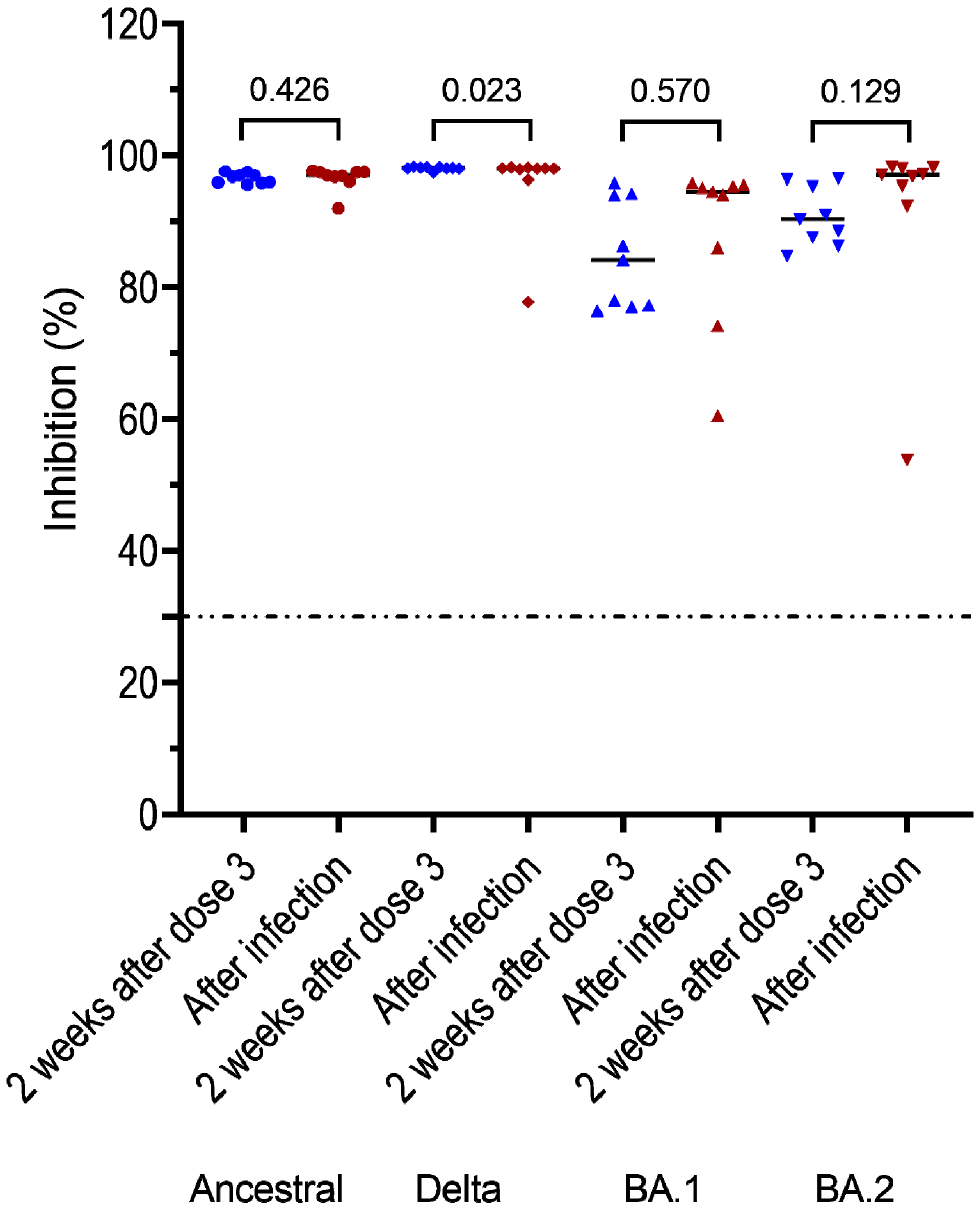

Supplementary Figure 2: Neutralizing antibodies measured at week 2 and 15 after booster vaccination in 11 HCWs with an infection episode documented after the booster dose

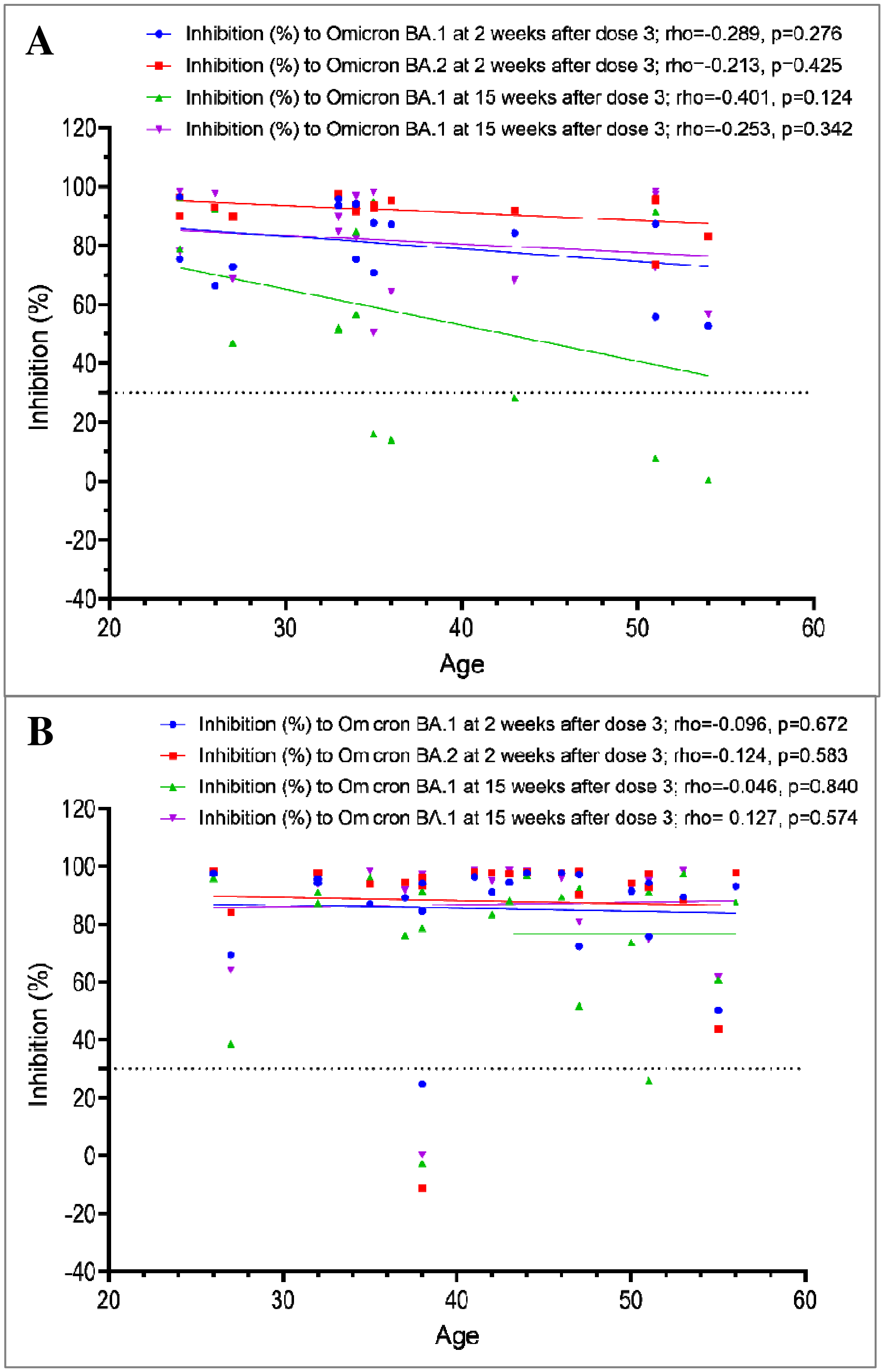

Supplementary Figure 3: Association between age and neutralizing antibody levels to BA.1 and BA.2 measured at week 2 and 15 post booster vaccination in those without a SARS-CoV-2 infection episode recorded after the booster dose, A): 16 individuals of G1 and B) 22 individuals of G2

Note to Supplementary Figure 3: rho: Spearman's rank correation coefficient
